# Colonization of plants by human pathogenic bacteria in the course of organic vegetable production

**DOI:** 10.3389/fmicb.2014.00191

**Published:** 2014-05-05

**Authors:** Andreas Hofmann, Doreen Fischer, Anton Hartmann, Michael Schmid

**Affiliations:** Department of Environmental Sciences, Research Unit Microbe-Plant Interactions, Helmholtz Zentrum München, German Research Center for Environmental Health (GmbH)Neuherberg, Germany

**Keywords:** organic food, vegetable, organic fertilizer, *Salmonella enterica*, *Listeria monocytogenes*

## Abstract

In recent years, increasing numbers of outbreaks caused by the consumption of vegetables contaminated with human pathogenic bacteria were reported. The application of organic fertilizers during vegetable production is one of the possible reasons for contamination with those pathogens. In this study laboratory experiments in axenic and soil systems following common practices in organic farming were conducted to identify the minimal dose needed for bacterial colonization of plants and to identify possible factors like bacterial species or serovariation, plant species or organic fertilizer types used, influencing the success of plant colonization by human pathogenic bacteria. Spinach and corn salad were chosen as model plants and were inoculated with different concentrations of *Salmonella enterica* sv. Weltevreden, *Listeria monocytogenes* sv. 4b and EGD-E sv. 1/2a either directly (axenic system) or via agricultural soil amended with spiked organic fertilizers (soil system). In addition to PCR- and culture-based detection methods, fluorescence *in situ* hybridization (FISH) was applied in order to localize bacteria on or in plant tissues. Our results demonstrate that shoots were colonized by the pathogenic bacteria at inoculation doses as low as 4 × 10 CFU/ml in the axenic system or 4 × 10^5^ CFU/g in the soil system. In addition, plant species dependent effects were observed. Spinach was colonized more often and at lower inoculation doses compared to corn salad. Differential colonization sites on roots, depending on the plant species could be detected using FISH-CLSM analysis. Furthermore, the transfer of pathogenic bacteria to plants via organic fertilizers was observed more often and at lower initial inoculation doses when fertilization was performed with inoculated slurry compared to inoculated manure. Finally, it could be shown that by introducing a simple washing step, the bacterial contamination was reduced in most cases or even was removed completely in some cases.

## Introduction

The consumption of vegetables is essential for a healthy nutrition and is recommended by different health organization in order to provide minerals and vitamins as well as for the prevention of cardiovascular diseases (World Health Organization, 2003; United States Department of Agriculture, 2011). In the years 1997–1999 an increase of the consumption of fresh vegetables was recorded in the USA, staying on this high level for the following years (Blanck et al., 2008; Berger et al., 2010). Most of this food is consumed raw or after minimal processing. Therefore, it is crucial to avoid its contamination with human pathogenic bacteria, viruses or health threatening substances throughout the production chain. Nevertheless, in the last years an increasing number of outbreaks caused by the consumption of vegetables contaminated with human pathogenic bacteria were reported (Sivapalasingam et al., 2004; Heaton and Jones, 2008). For example, in 2007 lettuce was the one of the three most frequent sources of foodborne disease outbreaks in the USA (Center of Disease Control and Prevention, 2010).

*Salmonella enterica* and *Listeria monocytogenes* are two foodborne human pathogens involved in many outbreaks. In the years 2002–2007 *S. enterica* was the most frequent causative agent of foodborne diseases in the USA. The largest outbreak in this time period with 802 documented patients was traced back to the consumption of “hummus” (mashed chickpeas) contaminated with this bacterium (Center of Disease Control and Prevention, 2010). Hanning et al. (2009) summed up *S. enterica* outbreaks in the USA based on the consumption of fresh produce showing that many different vegetable plant species were involved in those outbreaks. Due to its importance as produce contaminant much research has been done in this direction proving the high potential of *S. enterica* to colonize surfaces as well as interior of various plants (Brandl, 2006; Berger et al., 2010; Krtinić et al., 2010). *Listeria monocytogenes* outbreaks based on the consumption of fresh produce were reported in lower numbers compared to those derived from other food sources (US Food and Drug Administration, 2003). Nevertheless, a recent outbreak of listeriosis in the USA in 2011 was caused by the consumption of contaminated cantaloupes (Center of Disease Control and Prevention, 2011). Also in sporadic cases of infections with *L. monocytogenes*, vegetables were identified as causative food source (Farber and Peterkin, 1991). However, in a high number of outbreaks or single cases, no specific food source could be identified, because of the very long incubation time until appearance of listeriosis symptoms (McLauchlin, 1996). Therefore, the US food and drug administration (FDA) as well as the EU dictate a zero tolerance policy for *S. enterica* and *L. moncytogenes* in ready to eat food throughout the production chain, although the EU-legislation allows 100 CFU of *L. moncytogenes* in 25 g of sample material, for food already placed on the marked.

Various possibilities of contamination with human pathogenic bacteria exist within the vegetable production chain. For example, bacteria could be transferred to the plants by contaminated irrigation water, via soil or direct contact (Wachtel et al., 2002). Different wild animals were also proven to be possible carrier of various human pathogenic bacteria (Palmgren et al., 1997; Makino et al., 2000; Handeland et al., 2002; Millan et al., 2004; Renter et al., 2006; Hellström et al., 2008; Sánchez et al., 2010; Wacheck et al., 2010; Vieira-Pinto et al., 2011). It cannot be excluded that those animals can access the agricultural fields and directly contact the plants grown there. The fertilization with slurry or manure is an additional way of contamination (Chen and Jiang, 2014). It has been shown that organic fertilizers can contain various human pathogenic bacteria (Hutchison et al., 2004, 2005). A transfer via direct contact with the plants or indirectly via the fertilized soil is therefore possible. In order to enable a reduction of the pathogen load in the soil the US government dictates that organic fertilizer has to be incorporated not less than 120 days before harvest of plants. However, regulations regarding the time point of organic fertilizer application strongly differ between countries. Furthermore, contamination of produce may also occur in postharvest processing steps. For example *L. monocytogenes* was detected in samples of the food processing environment like door handles, floors and walls (O'Connor et al., 2010). Similar results for *S. enterica* were obtained in a comparable study (Lettini et al., 2012). Those bacteria can also form biofilms on stainless steel surfaces and by that be protected from disinfection (Sinde and Carballo, 2000).

Based on the hypothesis that most of the contaminations of vegetable plants by human pathogenic bacteria can effectively be reduced already at farm level, this study focuses on the first steps of the organic vegetable production chain at the farm level until harvest. In order to demonstrate the influence of plant host species and bacterial species on plant colonization success, the minimal bacterial infection doses of the selected bacterial strains *S. enterica* sv. Weltevreden, *L. monocytogenes* sv. 4b and *L. monocytogenes* EGD-E sv.1/2a needed for the colonization of spinach or corn salad plants were determined in inoculation experiments. The *S. enterica* sv. Weltevreden strain used in this study was originally isolated from an outbreak of salmonellosis caused by the consumption of contaminated alfalfa sprouts (Emberland et al., 2007). The *L. monocytogenes* serotypes 1/2a and 4b were selected because of their high clinical relevance as causative strains of a large number of listeriosis cases. To meet the zero tolerance policy in ready to eat foods throughout the production chain for the contamination with *L. monocytogenes* and *S. enterica*, another objective was to develop and apply a most sensitive and reliable combination of cultivation enrichment and PCR-detection approach for the studied pathogenic bacteria. Furthermore, the colonization sites of the inoculated bacterial species on plant roots were identified by combining fluorescence *in situ* hybridization (FISH) and confocal laser scanning microscopy (CLSM). The growth conditions in the axenic system as well as the absence of competition by soil bacteria might enhance bacterial colonization of plants (Klerks et al., 2007). Therefore, and in order to verify the results of the inoculation experiments, regarding the influence of plant- as well as bacterial species, in a more natural system, experiments in a soil system using spiked manure or slurry for fertilization of the plants were applied. By this, the influence of the type of organic fertilizer used, on plant colonization by the selected human pathogenic bacteria was analyzed. The results presented identified factors influencing the frequency of colonization of vegetable plants by human pathogenic bacteria. This may help to minimize the risk of produce contamination already at the farm level and thus may increase the safety for the consumer.

## Materials and methods

### Plant species and bacterial strains

Spinach (*Spinacia oleracea*) variety “Butterflay” and corn salad (*Vallerianella locusta*) variety “Verte á ceour plein 2” (Bingenheimer Saatgut AG, Echzell-Bingenheim, Germany) and three bacterial strains were used for inoculation experiments: *L. monocytogenes* EGD-E sv. 1/2a (DSM20600, Deutsche Stammsammlung von Mikroorganismen und Zellkulturen GmbH, DSMZ, Germany), *L. monocytogenes* sv. 4b (SLCC4013, special *Listeria* culture collection, Würzburg, Germany) and *S. enterica* sv. Weltevreden (2007-60-3289-, culture collection, zoonosis laboratory, DTU-FOOD, Denmark).

### Preparation of plant seedlings

Plant seeds were surface sterilized according to the protocol published by Rothballer et al. (2003) with slight modifications. The seeds were washed in 1% Tween80 (2 min) and subsequent in 70% ethanol (2 min) followed by three washing steps in sterile deionized water. After an incubation step in 13% sodium hypochlorite solution (Sigma-Aldrich® Co., St. Louis, USA) (20 min) seeds were again washed three times in sterile deionized water, incubated in sterile deionized water for 4 h followed by a second incubation step in 13% sodium hypochlorite solution for 10 min. Five washing steps in sterile deionized water completed the surface sterilization. Seeds were then placed on NB (Nutrient Broth) agar plates and incubated for 3 days at room temperature in the dark to allow germination and to control the success of surface sterilization. Only germinated seedling showing no visible contamination were used for further experiments.

### Inoculation experiments in an axenic model system

*Listeria monocytogenes* strains were cultivated in Brain-Heart-Infusion (BHI) liquid media at 30°C and *S. enterica* sv. Weltevreden in buffered peptone water (BPW) at 37°C over night. The bacterial cells were washed and afterwards diluted in 1 × PBS to a final density of 4 × 10^8^ CFU/ml. The germinated seedlings were inoculated for 1 h in different dilutions ranging from 4 × 10 to 4 × 10^6^ CFU/ml of the different bacterial strains. Non-inoculated seedlings were used as control. After inoculation seedlings were planted to sterile “Phytatray 2”-boxes (Sigma-Aldrich® Co., St. Louis, USA) filled with 20 ml sterile quartz sand and 10 ml MS (Murashige and Skoog) medium (Sigma-Aldrich® Co., St. Louis, USA). Three boxes with four seedlings per box were prepared for each inoculation dose. Plants were grown for 3 weeks in a phytochamber (humidity: 50%, day length: 14 h, day temperature: 23°C, night temperature: 18°C, light intensity: 360 μmol/m^2^s) until harvest.

### Spiking experiments in the soil system

Fresh, not processed organic bovine slurry and manure as well as organically managed agricultural soil (Ap horizon) obtained from the “Versuchsgut Scheyern” of the Helmholtz Zentrum München used in this study were initially tested for the presence of *L. monocytogenes* and *S. enterica* using enrichment as well as PCR-methods. Only organic fertilizer and soil considered free of those bacteria were used for further experiments. Bacterial strains were prepared as decribed above and added to slurry and manure at concentrations of 4 × 10^5^, 4 × 10^6^, 4 × 10^7^, and 4 × 10^8^ CFU/ml slurry or CFU/mg manure. Five hundred grams of fresh weight of agricultural soil were mixed with 100 ml deionized water and 20 g of spiked manure or 30 ml of spiked slurry and filled into planting pots. The amount of organic fertilizer used is in accordance to usual farming practice (2–4 kg/m^2^ for manure, 3–6 l/m^2^ for slurry). The final bacterial concentrations in the soil were 2.4 × 10^4^, 2.4 × 10^5^, 2.4 × 10^6^, and 2.4 × 10^7^ CFU/g for slurry setups and 1.6 × 10^4^, 1.6 × 10^5^, 1.6 × 10^6^, and 1.6 × 10^7^ CFU/g for manure. After a preincubation of the pots for 3 days at room temperature in a mini greenhouse (Edm. Romberg & Sohn GmbH & Co. KG, Ellerau, Germany), the sterile seedlings were planted in the pots. Three pots with four seedlings each were used per dilution step and incubated for 4 weeks in a phytochamber with the settings described above. Pots prepared with non-spiked organic fertilizer served as negative control.

### Sampling and sample preparation

Plant samples of the axenic system were taken in triplicates whereas five samples per treatment were taken from the soil system. After harvest, root and shoot of the plants were separated aseptically. The plant parts were washed in 30 ml of 1 × PBS and afterwards ground in 1 ml 1 × PBS. One milliliter of the resulting cell suspension was used for enrichment cultures. Also the 1 × PBS used for washing was further processed. After a cell harvest for 10 min at 6000 rpm the resulting pellet was resuspended in 2 ml of 1 × PBS. Again 1 ml of the cell suspension was used for further enrichment. Additionally, fixed samples of plant parts were prepared after cutting off parts of roots and shoots. *Salmonella enterica* inoculated or spiked samples were fixed in 4% paraformaldehyde (PFA) according to Amann et al. (1990), whereas *L. monocytogenes* inoculated or spiked samples were fixed in 1:1 Ethanol/PBS (Roller et al., 1994). Fixed plant samples were stored at −20°C in 1:1 Ethanol/PBS until further processing.

### Enrichment of the bacteria

For the selective enrichment of *Listeria* spp. 1 ml of cell suspension was added to 9 ml Buffered-*Listeria*-Enrichment-Broth (BLEB) (Merck KGaA, Darmstadt, Germany). After 2 h of preincubation at 30°C *Listeria*-Selective-Enrichment supplement (Merck KGaA, Darmstadt, Germany) was added and the culture was further incubated at 30°C. After 24 h, 48 h, and 7 days 0.1 ml of the enrichment culture were transferred to 10 ml Half-Fraser-Bouillon (Merck KGaA, Darmstadt, Germany) and incubated aerobically at 37°C. Enrichment culture dependent detection of *Listeria* spp. was conducted after 24 and 48 h using Oxford-Agarplates (Merck KGaA, Darmstadt, Germany) and Palcam-Agarplates (Merck KGaA, Darmstadt, Germany) inoculated with the second enrichment culture. In case of a detection of *Listeria* spp. Rapid‘L.mono medium (Bio-Rad Laboratories GmbH, Munich, Germany) plates were used to identify *L. monocytogenes*. After inoculation, the identification plates were incubated at 37°C for 48 h.

In order to enrich *S. enterica* cells, 1 ml of cell suspension was added to 9 ml of BPW and incubated aerobically 37°C for 24 h. Afterwards cultures were streaked out on Xylose-Lysine-Deoxycholat (XLD) agar plates (Fluka, Buchs, Switzerland) and incubated at 37°C for 24 h in order to identify *Salmonella* spp.

### DNA-extraction

DNA-extraction was conducted for *L. monocytogenes* after 48 h of enrichment in BLEB and for *S. enterica* after 24 h of enrichment in BPW. Cells of 2 ml enrichment culture were harvested at 6000 rpm for 10 min. *Salmonella enterica* samples were processed directly whereas the pellet *L. monocytogenes* enrichment cultures was resuspended in 50 μl of 300 U/ml Mutanolysin (Sigma-Aldrich® Co., St. Louis, USA) and incubated for 30 min at 37°C in order to increase the DNA-yield (Fliss et al., 1991). DNA-extraction was conducted using the “Bio 101 FastDNA® SPIN Kit for Soil” and the “Fast-Prep-Instrument” (MP-Biomedicals LLC., Solon, USA) following the manufacturers' instructions. Quality and quantity of the extracted DNA was analyzed using a ND-1000 Nanodrop-photometer (Thermo Fisher Scientific, Waltham, USA) and stored at −20°C until further processing.

### PCR-based detection of *L. monocytogenes* and *S. enterica*

The specific PCR-detection of *L. monocytogenes* was conducted using the *iap* targeted primerset MonoA/MonoB described by Bubert et al. (1992) and the detection of *S. enterica* by using the *inv-A* specific Primerset inv-Af/inv-Ar described by Rahn et al. (1992). The “Top Taq Polymerase” system (QIAGEN, Hilden, Germany) was applied for all PCR reactions following the manufacturers protocols. In order to increase specificity a “Touchdown-PCR” program (Don et al., 1991) was used. Therefore, the annealing temperature was lowered by 1°C every cycle starting from 70°C down to 63°C followed by 35 cycles with 63°C annealing temperature. The initial denaturation of 94°C for 5 min was followed by cycles comprised of a denaturation of 94°C for 30 s, an annealing for 30 s and an elongation of 72°C for 1 min. A final elongation step at 72°C for 10 min was integrated before samples were stored at 4°C. Success of the PCR-reaction and length of the amplification product was controlled by applying horizontal gelelctrophoresis. To verify the correct amplification of a positive PCR-signal the resulting fragments were purified using the NucleoSpin® Extract II Kit (Macherey & Nagel, Düren, Deutschland) and sequenced with a capillary sequencer ABI 3730 (Applied Biosystems) following the chain termination method (Sanger et al., 1977).

### Fluorescence *in situ* hybridization (FISH) and confocal laser scanning microscopy (CLSM)

FISH was conducted on root samples of plants grown in the axenic system, initially inoculated with 4 × 10^6^ CFU/ml of the respective bacteria. Since the plants were free of contamination, no species specific detection for *L. monocytogenes* or *S. enterica* was necessary and the application of bacteria-specific 16S-rRNA targeted EUB-338 probe mix (EUB-338 I Amann et al., 1990, EUB-338 II, and EUB-338 III Daims et al., 1999) was sufficient. FISH was conducted based on Manz et al. (1992) and Amann et al. (1992) with modifications. Hybridization was performed in a 2 ml tube as described by Grube et al. (2009). After dehydration in an ethanol-series of 50, 80, and 100% ethanol for 3 min each, 100 μl of hybridization buffer [360 μl of 5 M NaCl, 40 μl of 1 M Tris/HCl pH 8.0, 2 μl of 10% (m/v) SDS, 1.6 ml of deionized sterile water] as well as 10 μl of a 20 ng/μl probe solution was added to the fixed sample. The hybridization at 46°C for 2 h was followed by a stringent washing step for 15 min at 48°C in washing buffer [9 ml of 5 M NaCl, 1 ml of 1 M Tris/HCl pH 8.0, 50 μl of 10% (m/v) SDS, add 50 ml with sterile deionized water]. Afterwards, samples were placed on a microscope slide, embedded in Citifluor-A F1 (Citifluor Ltd., London, Großbritannien) and covered with a cover slid. CLSM analysis was performed using a LSM 510.meta (ZEISS, Oberkochen, Germany) equipped with a C-Apochromat® 63x/1.2 W Korr. objective. The “Zeiss LSM Image Browser version 4.2” software was used for image processing.

## Results

### Inoculation experiments in the axenic system

#### PCR and enrichment based detection of plant colonization

The detection of the inoculated bacteria was based on selective enrichment and specific PCR. In all samples qualified as “positive” the inoculated bacteria were detected with at least one of the applied methods. The results are summarized in Table 1.

**Table 1:**
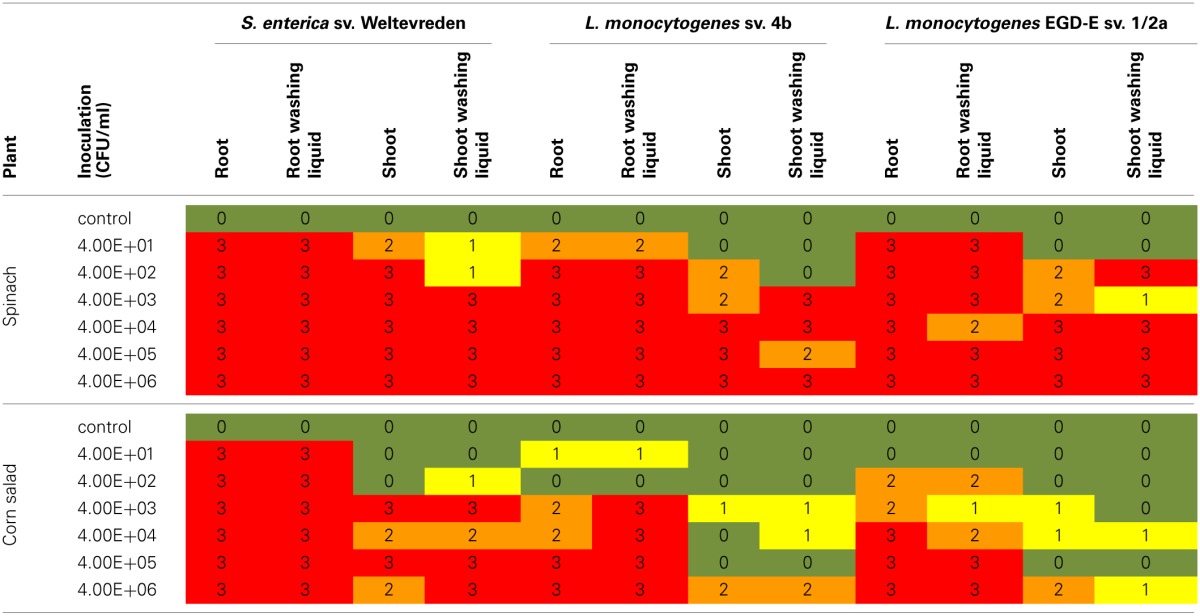
**Results of the inoculation experiment in the axenic system**.

Numbers and colors in the table indicate the numbers of positive detections of the bacterial strains of interest with at least one of the applied detection methods. For each dilution step three plants were analyzed.

In spinach and corn salad, *S. enterica* sv. Weltevreden was detected in all root samples and all samples of the root washing liquid. In spinach shoot and shoot washing liquid samples the bacteria also were found at initial inoculation doses as low as 4 × 10 CFU/ml. Nevertheless, in samples inoculated with 4 × 10 and 4 × 10^2^ CFU/ml, *S. enterica* sv. Weltevreden was detected more often in shoot plant samples compared to washing liquid samples. In contrast, corn salad shoots and the according washing liquids were found to be positive for the bacteria at inoculation doses of 4 × 10^3^ and 4 × 10^2^ CFU/ml, respectively.

In spinach plants, both of the used *L. monocytogenes* strains were found in root and root washing liquid samples at all inoculation doses. In samples inoculated with *L. monocytogenes* sv. 4b the lowest inoculation dose which resulted in a contamination of spinach shoot but not the according washing liquid could be observed was 4 × 10^2^ CFU/ml. Positive detection was possible in nearly all shoot and shoot washing liquid samples inoculated with higher bacterial doses. This was also found for *L. monocytogenes* EGD-E sv. 1/2a in samples inoculated with 4 × 10^2^ CFU/ml or more. In corn salad plants inoculated with *L. monocytogenes* sv. 4b only few root and root washing liquid samples were positive at inoculation doses of less than 4 × 10^3^ CFU/ml and only few shoots were colonized at inoculation doses below 4 × 10^6^ CFU/ml. The same was true for corn salad inoculated with *L. monocytogenes* EGD-E sv. 1/2a, with the exception that root colonization took already place at an inoculation dose of 4 × 10^2^ CFU/ml.

#### Combined FISH/CLSM analysis of inoculated roots

**Figure 1:**
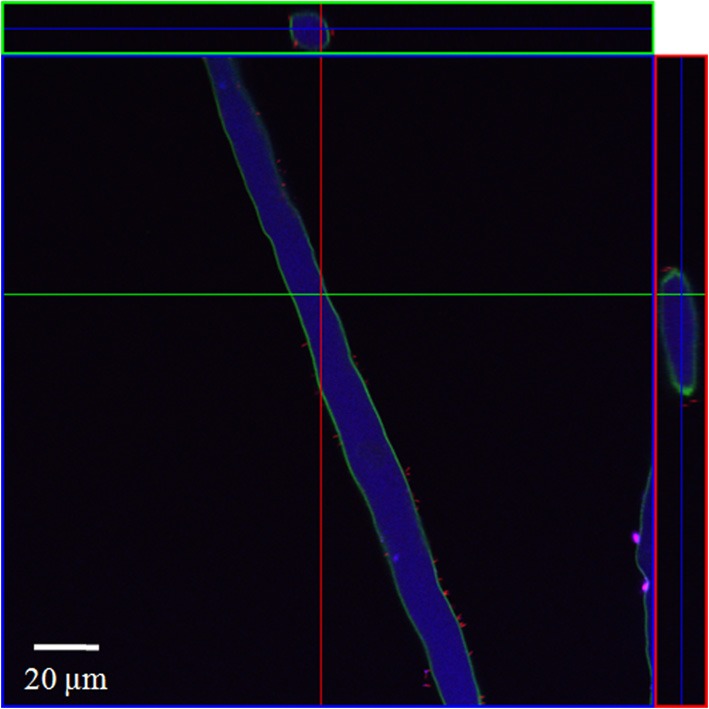
**CLSM image of a spinach root hair**. The plant seedling was inoculated for 1 h with *S. enterica* sv. Weltevreden at an inoculation density of 4 × 10^6^ CFU/ml. The plant was harvested after 3 weeks of growth in an axenic system. Probe Cy3 marked EUB-338, I, II, III was applied during FISH procedure (red signal).

**Figure 2:**
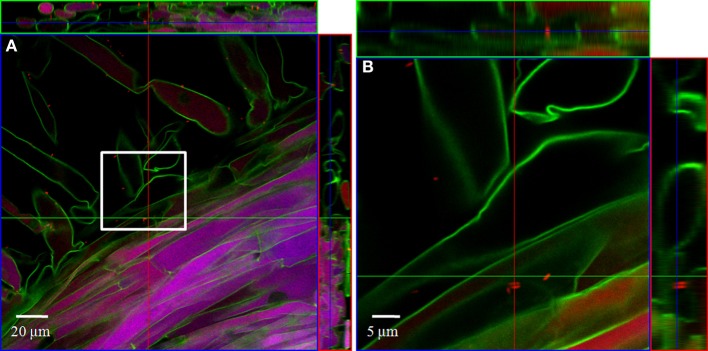
**CLSM images of a spinach root**. The plant seedling was inoculated for 1 h with *S. enterica* sv. Weltevreden at an inoculation density of 4 × 10^6^ CFU/ml. The plant was harvested after 3 weeks of growth in an axenic system. Probe Cy3 marked 338, I, II, III was applied during FISH procedure (red signal). **(B)** is an enlarged capture of the area marked in **(A)**.

**Figure 3:**
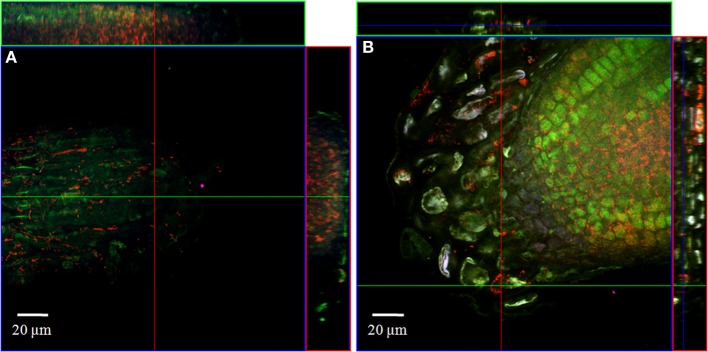
**CLSM images of corn salad root tips**. The plant seedlings were inoculated for 1 h with *S. enterica* sv. Weltevreden at an inoculation density of 4 × 10^6^ CFU/ml. The plants were harvested after 3 weeks of growth in an axenic system. Probe Cy3 marked EUB-338, I, II, III was applied during FISH procedures (red signal). The images show **(A)** surface colonization and **(B)** glycocalix colonization of the root tips.

**Figure 4:**
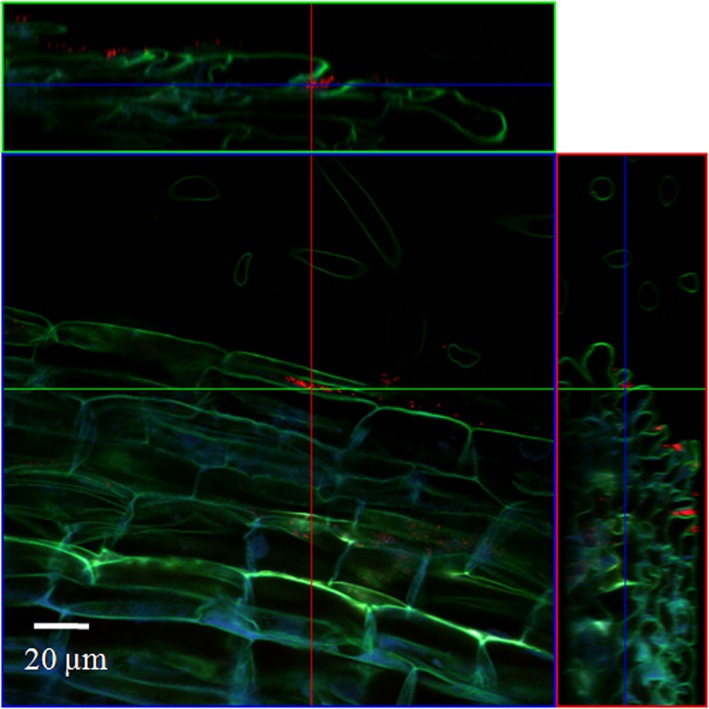
**CLSM image of a spinach root**. The plant seedling was inoculated for 1 h with *L. monocytogenes* sv. 4b at an inoculation density of 4 × 10^6^ CFU/ml. The plant was harvested after 3 weeks of growth in an axenic system. Probe Cy3 marked EUB-338, I, II, III was applied during FISH procedure (red signal).

**Figure 5:**
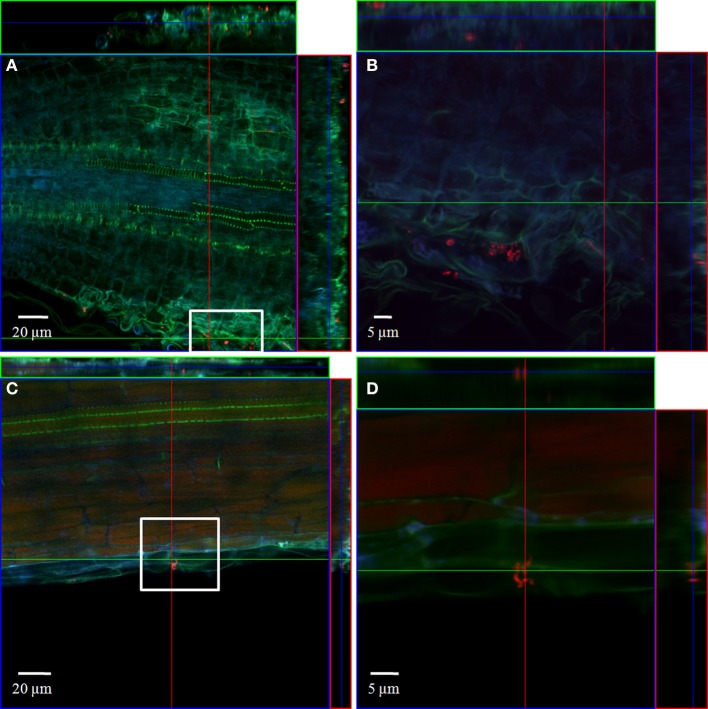
**CLSM images of a corn salad root tip (A,B) and a corn salad main root (C,D)**. The plant seedlings were inoculated for 1 h with *L. monocytogenes* sv. 4b at an inoculation density of 4 × 10^6^ CFU/ml. The plants were harvested after 3 weeks of growth in an axenic system. Probe Cy3 marked EUB-338, I, II, III was applied during FISH procedure (red signal). **(B)** is an enlarged capture of the area marked in **(A)**, **(D)** is an enlarged capture of the area marked in **(C)**.

In order to localize the inoculated bacteria on the roots of spinach and corn salad and to identify preferential colonization sites, a combined FISH/CLSM analysis was conducted. *Salmonella enterica* sv. Weltevreden was found to colonize spinach roots preferentially in the root hair zone. It was detected both, on the surface of the root hairs (Figure 1) as well as in cell interspaces of the main root in this zone (Figure 2). On corn salad roots the bacteria were mainly detected on the surface of root tip cells (Figure 3A) and rarely in the root hair zone (data not shown). On the root tip even a colonization of the glycocalix was observed (Figure 3B). *Listeria monocytogenes* sv. 4b colonized spinach roots in the root hair zone in cell interspaces of the root (Figure 4). It was not detected on root hairs or on the root tips (data not shown). Corn salad plants were mainly colonized by *L. monocytogenes* sv. 4b shortly behind the root tip but were not detected at the root tip (Figure 5). They were also found in cell interspaces of older root parts, but only in rare cases and small numbers (Figure 5).

### Spiking experiments in the soil system

The detection of *S. enterica* sv. Weltevreden was performed using *invA* gene specific PCR after enrichment in BPW, whereas colonization by the *L. monocytogenes* strains was analyzed using the *iap* gen targeted PCR as well as a selective enrichment. All obtained PCR-fragments were sequenced to avoid false positive results. In the samples qualified as “positive” at least one of the used methods of analysis was successful. The results are summarized in Table 2.

**Table 2:**
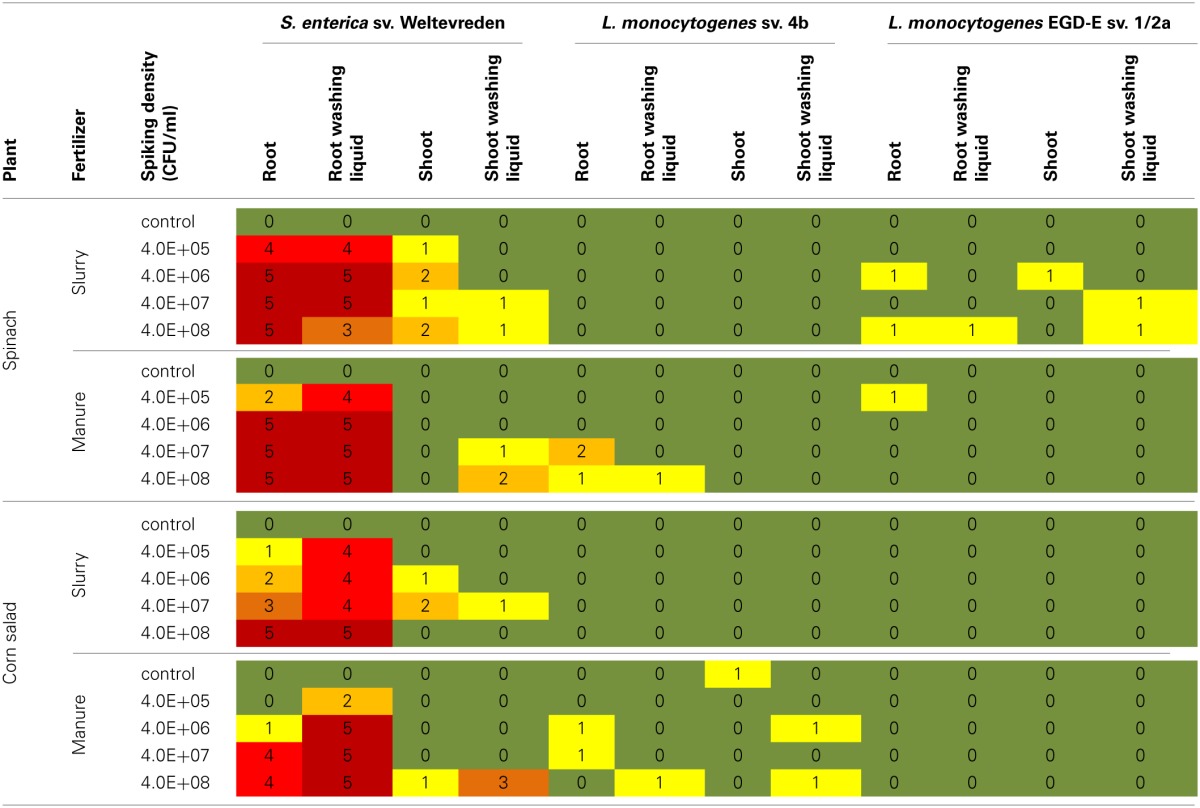
**Results of the spiking experiments in the soil system**.

Numbers and colors indicate the number of positive detection events. For each dilution step five plants were analyzed.

#### Colonization by S. enterica sv. weltevreden

Spinach roots were colonized by *S. enterica* sv. Weltevreden at all investigated spiking densities independent from the organic fertilizer used. Shoot plant samples of spinach were exclusively tested “positive” when fertilized with spiked slurry even at the lowest spiking density of 4 × 10^5^ CFU/ml. In contrast, in shoot washing liquid samples the bacteria were only detected at high spiking doses of at least 4 × 10^7^ CFU/ml, independent of the organic fertilizer used.

Corn salad roots were found to be colonized by the tested bacteria in samples of all spiking doses. More frequent positive detection events were observed in root washing liquid samples compared to the root samples. *Salmonella enterica* sv. Weltevreden was detected in three shoot samples fertilized with spiked slurry at spiking densities of 4 × 10^6^ and 4 × 10^7^ CFU/ml whereas only one washing liquid sample at a spiking density of 4 × 10^7^ CFU/ml was found to be positive. Corn salad shoots of plants fertilized with spiked manure were exclusively colonized by the bacteria of interest at the highest spiking dose of 4 × 10^8^ CFU/ml. Washing liquid samples also here were more often tested positive compared to plant samples.

#### Colonization by the L. monocytogenes serovariations

No bacterial colonization was found in plants when *L. monocytogens* sv. 4b spiked slurry was used and for corn salad amended with *L. monocytogenes* EGD-E sv. 1/2a spiked organic fertilizer. In spinach samples, fertilized with *L. monocytogenes* sv. 4b spiked manure, the bacteria of interest were only detected in root and root washing samples of the two highest spiking doses. For corn salad fertilized with spiked manure, only few samples independent of the spiking doses were tested positive. In this treatment even a contamination of a control plant was observed, which might be due to the fact that a contamination of soil or manure with *L. monocytogenes* cannot be excluded as those bacteria are very commonly found in this environments. Spinach fertilized with *L. monocytogenes* EGD-E sv. 1/2a spiked organic fertilizer was also only very rarely colonized.

## Discussion

The experiments presented in this study were conducted to identify the minimal infection doses of *S. enterica* sv. Weltevreden, *L. monocytogenes* sv. 4b, and *L. monocytogenes* EGD-E sv. 1/2 a needed for colonization of spinach or corn salad plants at axenic or at soil conditions. In order to achieve the highest sensitivity for the detection of a pathogen contamination, an enrichment step was used before DNA extraction and PCR analysis were performed. However, due to this enrichment step no complete quantitative analysis of the contamination was possible. In a similar experimental approach Arthurson et al. (2011) stated that the detection limit for *S. enterica* sv. Weltevreden is approximately 10^4^ CFU/g soil or plant material when quantitative PCR is applied. The same detection limit was reached for *L. monocytogenes* by Chen et al. (2011) when using direct PCR detection with different food matrices. In contrast, the authors were able to lower the detection limit down to 3 CFU/ml by introducing an enrichment step of 48 h in BLEB. Danyluk and Schaffner (2011) stated that on spinach plants *E. coli* O157:H7 cell numbers of 10^−1^ CFU/g on 0.1% of the plants at the time point of harvest are enough to possibly cause an outbreak. This assumption was made using a model, based on data, which revealed a strong growth of contaminant bacteria on harvested plant material if no cooling was applied (Abdul-Raouf et al., 1993; Chang and Fang, 2007; Lee and Baek, 2008). This demonstrated the need of a method with an extremely low detection limit, when analyzing human pathogenic bacteria on plants.

In addition to the highly sensitive detection, we also intended to localize the inoculated or spiked pathogens using fluorescence labeling of the bacteria by FISH and CLSM-analysis. Earlier studies have already shown that *S. enterica* can form biofilms on leaf surfaces (Kroupitski et al., 2009b) and even colonize the leaf interior via open stomata (Kroupitski et al., 2009a), which supports our findings in the axenic inoculation experiments. Especially in spinach, *S. enterica* sv. Wletevreden and *L. monocytogenes* sv. 4b were often detected in shoot samples, but could not be washed off. Although, it was not possible with FISH/CLSM combined analyses of plant shoot material to clearly identify bacterial cells (data not shown) due to the strong auto-fluorescent properties of the plant material (Amann et al., 1990; Cheng et al., 2001; Ongeng et al., 2013), a strong colonization of spinach shoots could be demonstrated with the PCR-technique. In contrast, in all our experiments using corn salad or *L. monocytogenes* EGD-E sv. 1/2a as inoculant, the bacteria were detected more often in washing liquid samples compared to the plant samples. In those cases it was even possible to remove the bacteria completely by the simple washing step. FISH/CLSM combined analysis of the root samples from the axenic system nevertheless revealed differential colonization sites depending on bacterial species as well as on plant species used for inoculation. Although no endophytic colonization which was observed for *S. enterica* and *E. coli* by Jablasone et al. (2005) was detected, the presence of the bacteria in cell interspaces of the root surface show a quite strong and persistent colonization of the plant roots.

The species or serovar of human pathogenic bacteria used for inoculation or spiking has a major influence on the extend of plant colonization. *Salmonella enterica* sv. Weltevreden was able to colonize the plants more efficiently and at lower initial inoculation or spiking dose compared to both serovariations of *Listeria monocytogenes*. This may be due to the fact that the strain used in this study was originally isolated from an outbreak traced back to the consumption of alfalfa sprouts (Emberland et al., 2007) and therefore was well adapted to colonize plants, in contrast to *L. monocytogenes* sv. 1/2a EGD-E which was isolated from rabbits (Murray et al., 1926) and *L. monocytogenes* sv. 4b isolated from a listeriosis patient (according to the Special *Listeria* Culture Collection, SLCC). Furthermore, it is known that *S. enterica* can be transferred to plants via contaminated organic fertilizer and soil (Klerks et al., 2007; Arthurson et al., 2011; Barak et al., 2011). In studies proving an endophytic colonization potential of *S. enterica, L. monocytogenes* was, in contrast, only detected on the plant surface (Jablasone et al., 2005; Kutter et al., 2006). Although being able to survive in soil (Botzler et al., 1974; Gorski et al., 2011) or manure amended soil (Jiang et al., 2004), *L. monocytogenes* was found to be inhibited by resident soil microbiota which can lead to a reduction of the cell numbers (McLaughlin et al., 2011). All this support our findings, that *L. monocytogenes* was only in few cases able to colonize plants in the soil system whereas colonization of plants grown in the axenic system was observed at even very low inoculation doses. Although differences in colonization success and interaction of some *Salmonella* spp. strains with lettuce cultivars have already been identified (Franz et al., 2007; Klerks et al., 2007), more research on the mechanisms of plant contamination by human pathogenic bacteria, including a higher number of bacterial strains is needed to clearly identify the reasons for the observed differences in the colonization success.

Furthermore, the plant species plays a crucial role in the colonization success by human pathogenic bacteria. *Salmonella enterica* and both *L. monocytogenes* serovariations were detected more frequently and at lower initial inoculation dose in spinach samples grown in the axenic system compared to corn salad plants. The same was true for the spiking experiments with *S. enterica*. Literature supports those findings. Yadav et al. (2005) for example showed that bacterial colonization of leaf surfaces strongly depends on the properties of those leaves, like water and phosphorus content or leafs and mesophyll thickness. Zhang et al. (2010) detected significantly different bacterial communities on leafs of different vegetable plant species. Even a plant cultivar based effect on the colonization of lettuce by *S. enterica* was proven by Klerks et al. (2007).

The type of organic fertilizer used also influences the colonization of the plants by the human pathogenic bacteria used. *Salmonella enterica* was detected more often and at lower spiking doses if the plants were grown in slurry amended soil. The reduction or removal of the plant contamination with simple washing of the vegetables was also less successful in those samples. Hutchison et al. (2004) found a direct influence of humidity and severity of pathogen contamination in a large sampling campaign of organic fertilizers. The higher humidity of the slurry compared to manure might therefore enhance survival of the pathogenic bacteria first in the organic fertilizer and later in soil. This is supported by findings of Semenov et al. (2009) showing that *S. enterica* as well as *E. coli* display an enhanced survival rate in slurry amended soil compared to manure amended soil due to the higher content of dissolved organic nitrogen and carbon in the slurry.

## Conclusions

De Roever (1998) formulated research needs to improve safety of fresh produce for the consumer. One of the key issues, which were also addressed in our study, is to improve farming practice in order to decrease the potential for produce contamination. We were able to show that this potential is strongly depending on the plant species under consideration. Therefore, “high risk plants,” in our case spinach, need to be treated with more care during production, harvest and processing. Furthermore, the type of organic fertilizer used also influences the colonization success. In order to minimize the risk of transfer of human pathogenic bacteria to produce, manure instead of slurry should preferably be used. Fermentation or composting increased the safety of the fertilizer by minimizing the number of pathogenic bacteria present (Vinnerås, 2007; Abdel-Mohsein et al., 2010). Plant roots were usually colonized more frequently compared to shoots. Therefore, co-harvesting of roots should be avoided as much as possible. Although the pathogenic bacteria were shown to colonize the plant surface, the possibility of an endophytic colonization, especially for *S. enterica* sv. Weltevreden cannot be excluded. We demonstrated that washing was suitable to reduce or even remove the bacterial contamination in most cases. Since the bacteria were frequently detectable in the washing liquid, it should not be reused. Washing of vegetable plants is therefore useful but should be performed under flowing water. By this simple means, the risk of contamination of produce with human pathogenic bacteria can already be reduced on farming level, which increases safety for the final consumer.

### Conflict of interest statement

The authors declare that the research was conducted in the absence of any commercial or financial relationships that could be construed as a potential conflict of interest.
